# Serum level of soluble interleukin 6 receptor is a useful biomarker for identification of treatment‐resistant major depressive disorder

**DOI:** 10.1002/npr2.12100

**Published:** 2020-03-12

**Authors:** Katsuhiko Yamasaki, Tomonori Hasegawa, Masatoshi Takeda

**Affiliations:** ^1^ Department of Neuropsychiatry The Institute of Health and Welfare Kensho‐kai Medical Corporation Osaka Japan; ^2^ SRL Inc. Tokyo Japan; ^3^ Cognitive Reserve Research Center Osaka Kawasaki Rehabilitation University Osaka Japan; ^4^ Jinmeikai Research Institute for Mental Health Hyogo Japan

**Keywords:** cytokines, IL‐6 trans‐signaling system, major depressive disorder, soluble IL‐6 receptor, treatment‐resistant depression

## Abstract

**Aim:**

A substantial proportion of major depressive disorder patients are treatment‐resistant to antidepressant therapy, who require augmentation drugs, or other treatments including electroconvulsive therapy or transcranial magnetic stimulation. Identifying treatment‐resistant major depressive disorder patients before the actual administration of antidepressant is, however, often difficult. Accordingly, the serum biomarker to identify treatment‐resistant patients will be helpful in clinical settings. This study aims to clarify the appropriate biomarkers for identification of treatment‐resistant major depressive disorder.

**Method:**

Given that immune‐inflammatory processes are involved in the pathogenesis of major depressive disorder, it is possible that certain cytokine‐related molecules could serve as clinically useful biomarkers of treatment‐resistant major depressive disorder patients. In this study, we measured serum levels of tumor necrosis factor‐α, interleukin 6, and soluble interleukin 6 receptor after major depressive disorder patients underwent antidepressant therapy.

**Results:**

The serum level of soluble interleukin 6 receptor, but not interleukin 6 or tumor necrosis factor‐α, was significantly higher in treatment‐resistant major depressive disorder patients than in remitted patients, suggesting that serum soluble interleukin 6 receptor could be a good biomarker of treatment‐resistant major depressive disorder. Receiver operating characteristic analysis confirmed that serum soluble interleukin‐6 receptor level measurement was useful for identification of treatment‐resistant major depressive disorder patients. Multiple regression analysis using the serum levels of the aforementioned cytokines as explanatory variables and the Quick Inventory of Depressive Symptomatology‐Self Report score (QIDS‐SR_16_) as a target variable showed that only serum soluble interleukin‐6 receptor level could explain the severity of major depressive disorder.

**Conclusion:**

Based on these results, we recommend measurement of serum soluble interleukin‐6 receptor level to discriminate treatment‐resistant major depressive disorder patients. High serum soluble interleukin‐6 receptor level is associated with the pathogenesis of treatment‐resistant major depressive disorder, suggesting the involvement of the interleukin 6 trans‐signaling system in onset of treatment‐resistant major depressive disorder.

## Abstract 2

Major depressive disorder is driven by immune‐inflammatory pathways. Treatment‐resistant cases are common in clinical settings, and the identification of cytokines associated with treatment‐resistant major depressive disorder could be useful targeting patients positive for these biomarkers. We found the serum level of soluble interleukin 6 receptor, but not interleukin 6 or tumor necrosis factor‐α, was significantly higher in treatment‐resistant than in remitted major depressive disorder patients, suggesting that serum soluble interleukin 6 receptor could be a useful biomarker for treatment‐resistant major depressive disorder.

## Graphical abstract

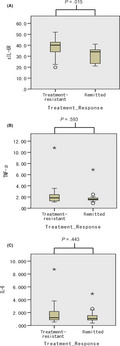

## 1 INTRODUCTION

Major depressive disorder (MDD) is a leading cause of disability with the highest disability‐adjusted life year (DALY) value among all diseases and disorders, and thus places a significant burden on society.^1^, ^2^ A substantial proportion of MDD patients are treatment‐resistant, that is, they do not enjoy satisfactory therapeutic outcomes in response to first‐line or subsequent attempts at pharmacological therapies.^3^, ^4^ Hence, more efficacious treatment for such patients is urgently required.

The definition of treatment‐resistant depression is controversial. In actual clinical settings, patients who receive antidepressants are evaluated for therapeutic outcome using rating scales based on subjective indexes; the relationships between the answers to each question are relative, and some degree of arbitrariness cannot be entirely eliminated. Given the importance of highly reproducible means for evaluating the effect of longitudinal treatment, more objective measures must be developed to evaluate the outcome of pharmacotherapy in MDD patients.

Recently, it has become clear that the pathogenesis of MDD involves immune‐inflammatory processes, and the cytokines interleukin‐6 (IL‐6) and tumor necrosis factor‐α (TNF‐α) have been implicated in induction of depressive symptoms.^5^, ^6^, ^7^, ^8^, ^9^ In addition, soluble IL‐6 receptor (sIL‐6R), which is produced by cleavage before the membrane‐spanning region of IL‐6 receptor,^10^, ^11^ may also play a role in the onset of depressive symptoms.^12^, ^13^ We investigated the usefulness of the serum levels of IL‐6, TNF‐α, and sIL‐6R as biomarkers for discriminating treatment‐resistant MDD patients from remitted patients.

The results revealed that serum sIL‐6R level is a biomarker of treatment‐resistant MDD. Because sIL‐6R can stimulate trans‐signaling by forming a ligand‐receptor complex with IL‐6 after binding to gp130, our findings suggest a possible role for the IL‐6 trans‐signaling system in the onset of treatment‐resistant MDD.

## 2 METHODS

### 2.1 Definition of treatment‐resistant and remitted MDD patients

A definition of treatment‐resistant MDD patient is crucial for selection of the study population. Treatment resistance can be partial or full, and the definition should rely on an operational algorithm. It is not currently known how many adequately delivered antidepressant trials are necessary in order to declare a patient treatment‐resistant. Modest resistance involves inadequate response to a single antidepressant trial, whereas greater resistance involves failures of two monotherapy trials or one or more augmentation trials.^14^, ^15^, ^16^ However, in usual clinical settings, if an episode of depression is not adequately treated by at least two adequate trials of medications from different classes of antidepressants, treatment resistance can be clinically declared. In this study, patients who had not achieved remission after receiving two or more medications from different classes of antidepressants for at least 12 weeks were defined as treatment‐resistant. On the other hand, remitted patients were defined as patients who had attained remission after receiving one or two antidepressant medication. A score of ≤5 on the Quick Inventory of Depressive Symptomatology‐Self Report (QIDS‐SR_16_) equivalent to ≤7 on the 17‐item Hamilton Rating Scale for Depression (HRSD_17_) was defined as remission.

### 2.2 Study design

From September 2018 to December 2018, blood samples were taken from patients who had been diagnosed with MDD without psychotic features according to the Diagnostic and Statistical Manual‐5 (DSM‐5^®^) criteria^17^ and were retrospectively judged to be treatment‐resistant or remitted at the point of blood sampling. To ensure clear results, remitted patients were restricted to those who exhibited no relapse from initial treatment until enrollment in this study. Patients were 20‐80 years of age, not pregnant, and not breast‐feeding. Patients with diagnosis of bipolar or psychotic disorders, obsessive‐compulsive disorders, eating disorders; a history of epilepsy, major medical and neurologic disorders, or drug or alcohol dependency or abuse were excluded from the study. Patients with inflammatory conditions such as rheumatoid arthritis or Hashimoto's disease were also excluded.

This research was conducted in accordance with the Declaration of Helsinki (1989). The study was reviewed and approved by Clinical Research Ethical Committee of Kensho‐kai Medical Corporation (No. 1201‐05‐005708). The patients/participants provided their written informed consent to participate in this study.

### 2.3 Biological measures

Blood was collected in 5‐mL EDTA‐containing vacutainers for measurement of IL‐6 and TNF‐α, and agar‐based vacutainers for measurement of sIL‐6R. Assays were performed at SRL for sIL‐6R and LSI Medience for IL‐6 and TNF‐α. Samples were centrifuged for 5 minutes at 1500 *g*, and the plasma was aliquoted and stored at −80°C until analysis. Blood samples were refrigerated at 4°C until processing. QuantiGlo ELISA Human IL‐6 Immunoassay, QuantiGlo Human TNF‐α Chemiluminescent Immunoassay 2nd generation, and Quantikine Human IL‐6 sR Immunoassay were used for IL‐6, TNF‐α, and sIL‐6R, respectively. The normal ranges of serum levels of IL‐6, TNF‐α, and sIL‐6R are ≤2.41 pg/mL, ≤1.79 pg/mL, and 14‐46 ng/mL, respectively.

### 2.4 Statistical analysis

Serum levels of TNF‐α, IL‐6, and sIL6‐R were compared between treatment‐resistant and remitted patients. Differences among groups were analyzed by IBM SPSS. Data are presented as mean ± SEM *P* < .05 was considered to represent a statistically significant difference.

Receiver operating characteristic (ROC) analysis was performed; a criterion of area under the curve (AUC) > .7 was used to determine treatment efficiency.

Multiple regression analysis was performed in SPSS by a stepwise selection method in which QIDS‐SR_16_ score was used as a dependent variable, and individual cytokine levels were used as independent variables. Correlation analysis was also performed between serum sIL‐6R level and each category of QIDS‐SR_16_ score, and factor analysis was performed based on the correlation‐coefficient matrix among categories of QIDS‐SR_16_.

## 3 RESULTS

A total of 25 treatment‐resistant and 17 remitted patients were enrolled in the study. The proportion of women was 52.0% among treatment‐resistant patients and 64.7% among remitted patients, with average ages of 52.4 ± 2.0 years and 53.6 ± 2.0 years, respectively. Patient characteristics did not differ significantly between treatment‐resistant and remitted patients (Table 1).

**Table 1:** Characteristics of treatment‐resistant and remitted patients

	Treatment‐resistant MDD patients n = 25	Remitted patients n = 17	*P* value*
Gender Female (%)	13 (52.0%)	11 (64.7%)	
Age Mean age (±SEM)	52.4 (50.4‐54.4)	53.6 (51.6‐55.6)	.784
BMI Mean BMI (±SEM)	24.2 (23.7‐24.7)	22.7 (21.9‐23.4)	.110
Duration from initial treatment Mean (±SEM)	40.6 (36.8‐44.4)	36.6 (33.3‐39.9)	.624
Baseline depression severity Mean (±SEM)	18.5 (18.1‐18.9)	16.7 (16.2‐17.2)	.067
Depression severity at experiment Mean (±SEM)	14.0 (13.3‐14.7)	3.1 (2.8‐3.3)	<.001

* *P* value of the difference between treatment‐resistant and remitted patients.

**Figure 1:**
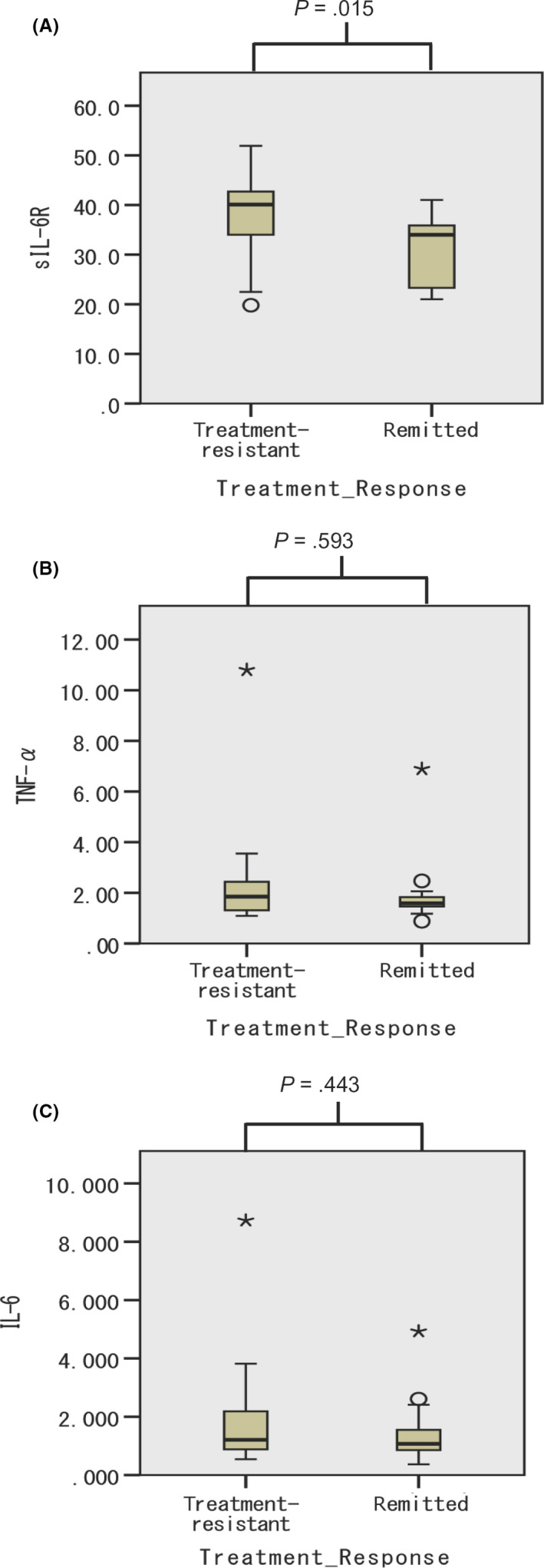
Serum concentration of each cytokine is shown as a box‐and‐whisker plot. The middle line in the box represents the median; the upper and lower lines represent the upper and lower quartile, respectively; and the upper and lower ends of the whiskers represent maximum and minimum values, respectively. Outliers are shown by ★, ○

Serum sIL‐6R level was significantly higher in treatment‐resistant patients (37.6 ng/mL, 95% confidence interval [CI] 34.0‐41.2 ng/mL) than in remitted patients (31.1 ng/mL, 95% CI 27.5‐34.6 ng/mL) (Figure 1A).

By contrast, there was no significant difference in serum TNF‐α level between treatment‐resistant (2.23 pg/mL, 95% CI 1.44‐3.02 pg/mL) and remitted patients (1.90 pg/mL, 95% CI 1.21‐2.58 pg/mL) (Figure 1B). Likewise, there was no significant difference in serum IL‐6 level between treatment‐resistant (1.79 pg/mL, 95% CI 1.11‐2.48 pg/mL) and remitted patients (1.41 pg/mL, 95% CI 0.85‐1.97 pg/mL) (Figure 1C).

**Figure 2:**
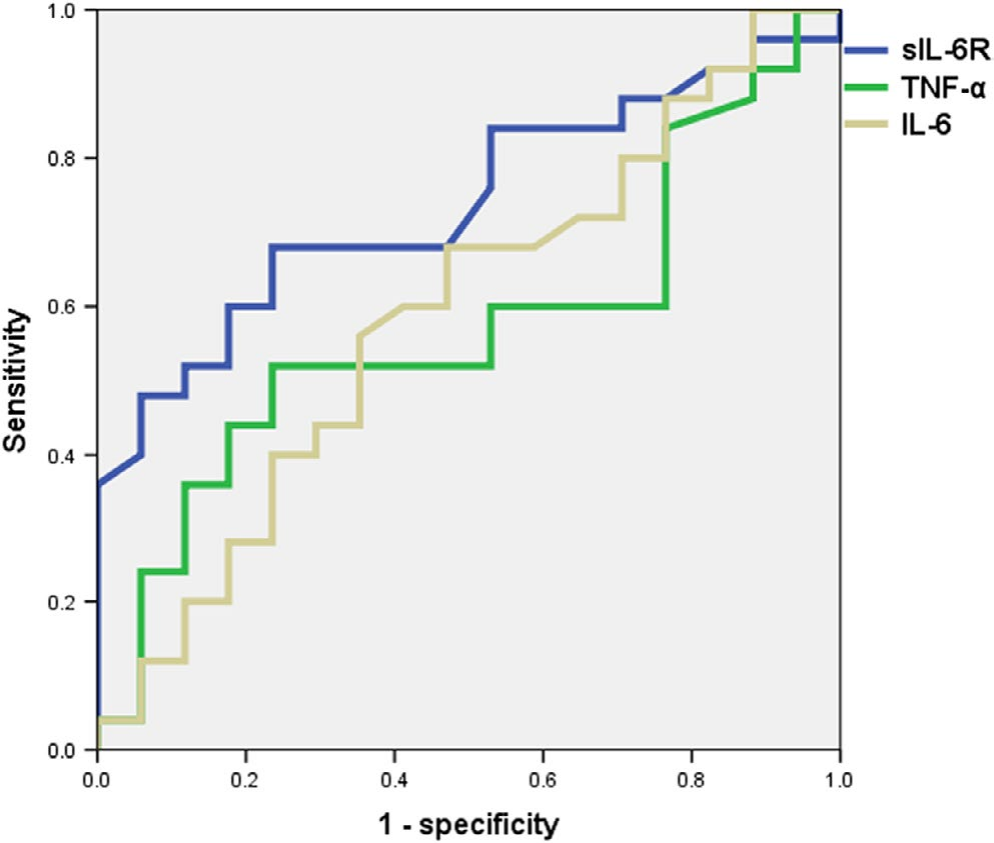
ROC analysis. AUC values were 0.739 (*P* = .009), 0.572 (*P* = .434), and 0.591 (*P* = .324) for sIL‐6R, TNF‐α, and IL‐6, respectively. Null hypothesis is that AUC is 0.5

In ROC analysis, serum sIL‐6R level yielded the only significant AUC (=0.789), which was the highest among the cytokines measured (Figure 2). Based on this analysis, the cutoff serum sIL‐6R level for discriminating treatment‐resistant from remitted patients was 35.80 ng/mL, with sensitivity and specificity of 0.68 and 0.71, respectively.

**Figure 3:**
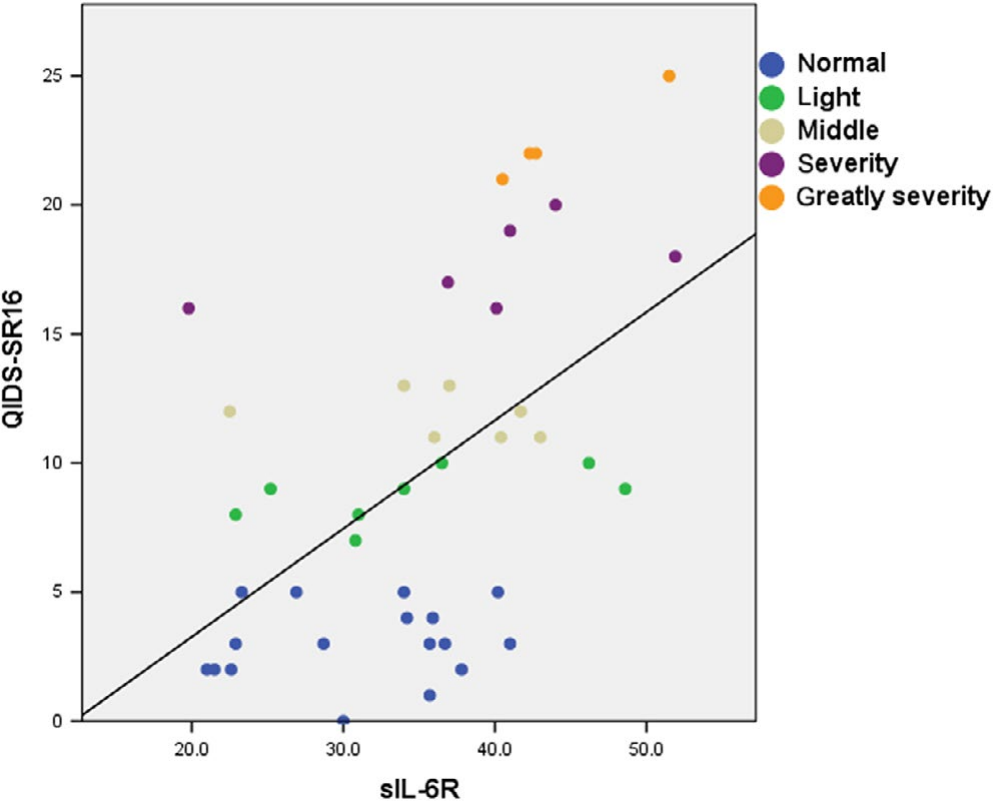
Correlation diagram. Regression equation was obtained as QIDS‐SR_16_ score = 0.420 × [concentration of sIL6‐R] − 5.123

**Figure 4:**
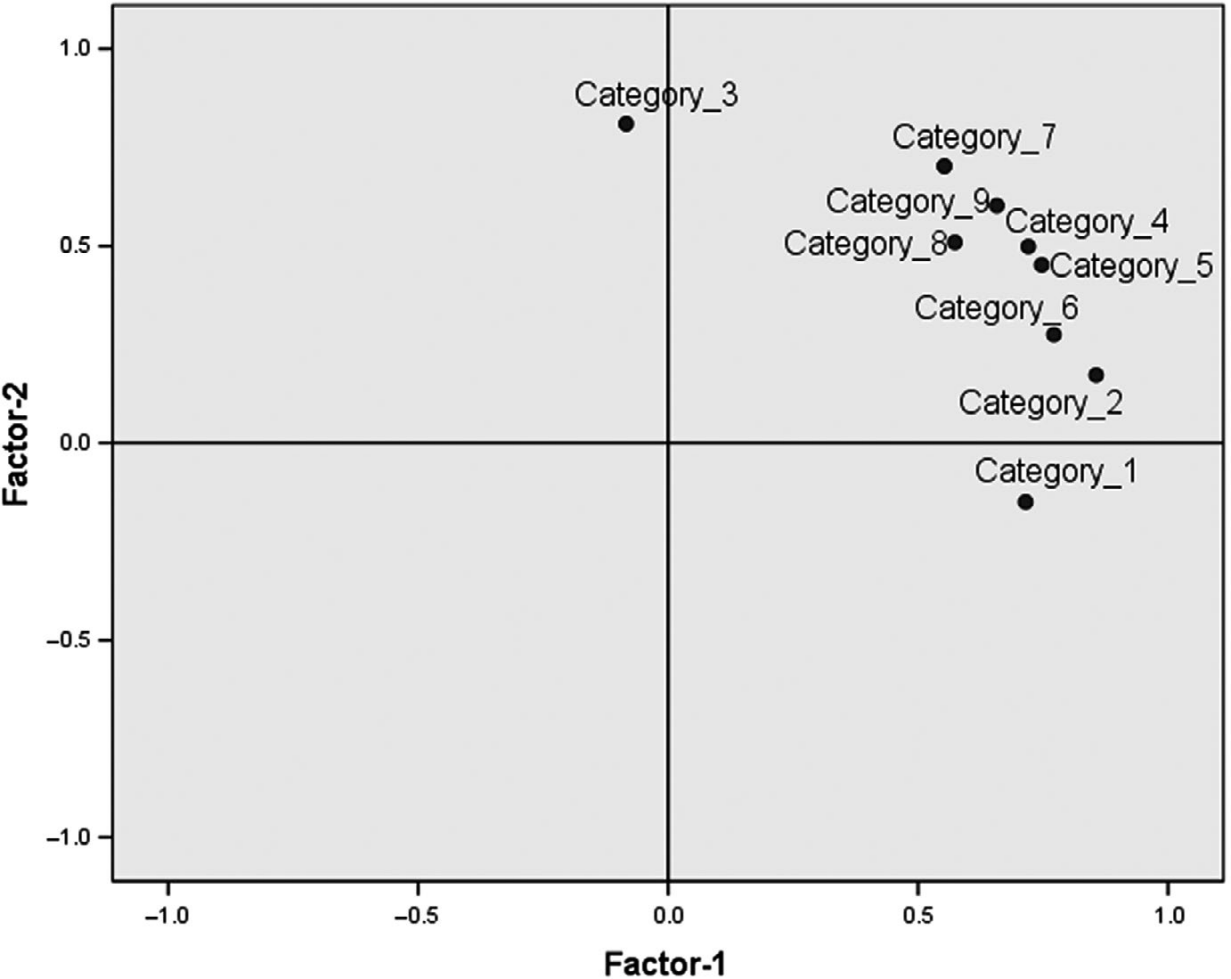
Factor plot expressed in two dimensions after varimax rotation. Two factors were extracted by principal component analysis. Category definitions are listed in the caption of Table 2

In multiple regression analysis, only sIL‐6R was extracted as an explanatory variable for QIDS‐SR_16_ score (Figure 3). The coefficient of determination was 0.278, which was not high, so we performed a correlation analysis between the score in each QIDS‐SR_16_ category and serum sIL‐6R level. We detected a significant correlation between serum sIL‐6R level and scores related to abnormality of appetite and loss of interest (Table 2). Sorrowful feelings and suicidal ideation had low correlation coefficients with serum sIL‐6R level, and the two categories were plotted near each other in the factor analysis (Figure 4).

**Table 2:** Correlation coefficient and *P* value for concentration of each cytokine and score of each of QIDS‐SR_16_

	Category 1	Category 2	Category 3	Category 4	Category 5	Category 6	Category 7	Category 8	Category 9
Correlation coefficient									
sIL‐6R	.053	.193	.525	.465	.421	.353	.550	.391	.447
TNF‐α	.063	.030	.174	.008	−.109	−.043	−.128	−.012	.074
IL‐6	.078	−.142	−.069	−.085	.053	−.164	−.057	−.079	.045
Significant probability									
sIL‐6R	.371	.110	<.001	.001	.003	.011	<.001	.005	.002
TNF‐α	.347	.424	.135	.480	.247	.393	.210	.471	.322
IL‐6	.313	.186	.332	.296	.369	.149	.359	.309	.390

Note

Category: Category 1, insomnia; Category 2, sorrowful feelings; Category 3, abnormality of appetite; Category 4, decrease in concentration; Category 5, view of self; Category 6, thought of death or suicide; Category 7, loss of interest; Category 8, energy level; Category 9, feeling slowed down or restless.

## 4 DISCUSSIONS

The number of MDD patients has been increasing around the world, causing substantial impairment of individual patients’ functions, as well as a significant economic burden to society.^18^ Most MDD patients are treated with antidepressants, including selective serotonin reuptake inhibitors (SSRIs) and serotonin‐noradrenaline reuptake inhibitors (SNRIs), but the efficacy of these medications is not ensured. Treatment‐resistant MDD patients are a significant subpopulation in normal clinical settings and require the development of new treatment methods.

Here, we demonstrated that serum sIL‐6R concentration is significantly higher in treatment‐resistant than remitted MDD patients. Our results show that serum sIL‐6R level is a potentially useful biomarker for identifying treatment‐resistant patients. In the future, physicians could develop effective treatments through experiments targeting patients with higher serum concentrations of sIL‐6R.

In regard to the underlying mechanisms of MDD, depressive symptoms are associated with sustained activation of innate immune system, leading to increased production of proinflammatory cytokines.^19^, ^20^, ^21^ Proinflammatory cytokines such as IL‐6 and TNF‐α contribute to serotonergic and noradrenergic dysfunction;^22^, ^23^, ^24^ increase glucocorticoid resistance through hypothalamic‐pituitary‐adrenal axis (HPA axis);^25^ activate microglial cells;^26^ and stimulate pathological synaptic pruning, which may induce structural and functional brain changes,^27^ ultimately leading to depressive symptoms and maladaptive behaviors.^28^, ^29^ The IL‐6 trans‐signaling system is involved in IL‐6 production in microglia and neurons; consistent with this, lipopolysaccharide injection can induce maladaptive behaviors.^12^

Inflammatory cytokines including IL‐6 are known to contribute to the pathogenesis of rheumatoid arthritis, and monoclonal antibody such as tocilizumab is effective to some patients but not all rheumatoid arthritis with high remission rate. It is essential to identify the rheumatoid arthritis patients who will respond to anti‐IL‐6 therapy. There are papers reporting biomarkers to identify those rheumatoid arthritis patients with good response using sIL‐6R levels, in which serum sIL‐6R level, but not IL‐6 level, reportedly predicts clinical remission in patients with rheumatoid arthritis treated with IL‐6 receptor antibody (tocilizumab).^30^, ^31^

Our finding of the higher serum sIL‐6R level of treatment‐resistant MDD patients may share the similar inflammatory process caused by cytokines including IL‐6 in rheumatoid arthritis and MDD. Because IL‐6 trans‐signaling causes microglial activation in the brain,^32^ it is possible that a high serum sIL‐6R level leads to the onset of treatment‐resistant MDD.

Khandaker et al reported the association between a functional IL‐6R genetic variant and risk of depression, suggesting *IL‐6R* Asp358 Ala variant is associated with decreased risk of severe depression causing decreased serum CRP level in spite of the increased serum IL‐6 level, consistent with an anti‐inflammatory effect downstream of IL‐6. They concluded that the IL‐6/ IL‐6R pathways are involved with pathogenesis of severe depression.^33^ Further study is required to determine the threshold serum sIL‐6R level necessary for treatment resistance. Since inflammatory process is regarded as the common process between MDD and somatic diseases including rheumatoid arthritis and cardiovascular diseases,^34^ we think our finding of higher serum sIL‐6R level with treatment‐resistant MDD patients is indicating the common pathogenetic process between MDD and somatic diseases.

This study has several limitations. Firstly, we do not have enough data of serum CRP to discuss the validity of the classification of inflamed depression and noninflamed depression based on serum CRP levels proposed by Khandaker.^35^ Our preliminary data show there is no significant difference between remitted and treatment‐resistant patients. This may be because that the serum CRP, as an acute phase protein, had already decreased at the point of blood sampling, or treatment‐resistant MDD patients are dependent to sIL‐6R trans‐signaling, which might affect the brain function without peripheral proinflammatory process oriented with higher serum IL‐6 and sIL‐6R together with higher CRP. We cannot conclude whether the treatment‐resistant MDD patients classified in our study are matching with the low‐grade systemic inflammation as reflected by elevated CRP.^35^ We do not know how much serum sIL‐6R penetrates the blood‐brain barrier, whether sIL‐6R concentrations in the brain and periphery are correlated, or whether they are regulated through the same or similar mechanisms.

Another limitation is that the coefficient of determination by regression analysis between serum IL‐6R level and QIDS‐SR_16_ score was not high. However, the correlations between serum IL‐6R level and certain categories’ score (abnormality of appetite and loss of interest) were significant. The reason why the coefficient of determination was not high is that QIDS‐SR_16_ includes categories (eg, sorrowful feeling and suicidal ideation) that might be correlated with another explanatory variable. Serum level of sIL6‐R eliminates the vulnerability of rating scales based on subjective self‐reporting (ie, bias among categories) due to difference between individuals, questions, and patients’ conditions. In this sense, it could be said that serum level of sIL‐6R is a novel biomarker that could be used as a universal objective index.

The third limitation relates to the change in cytokine levels after successful treatment. Dahl et al^36^ reported that serum cytokines levels were reduced after recovery. In this study, however, we detected no significant differences in serum TNF‐α level between remitted and treatment‐resistant patients. These results imply that IL‐6 trans‐signaling is relevant to the process downstream of the effect of TNF‐α.

It was not clear whether the serum sIL‐6R level of treatment‐resistant MDD patients was already high before MDD onset, or instead increased after the onset of MDD and persists in the high range, even after proper treatment. To address this point, it will be necessary to longitudinally monitor the serum sIL‐6R level from the beginning of MDD treatment.

## CONFLICT OF INTEREST

The authors declare no conflict of interest.

## AUTHOR CONTRIBUTIONS

KY involved in conception and design of the study, acquisition and analysis of data, drafting the manuscript and figures, and project administration. TH involved in conception and design of the study, analysis of data, and drafting the figures. MT: involved in supervision and validation.

## DATA REPOSITORY

All relevant data are included in [Link], [Link], [Link].

## APPROVAL OF THE RESEARCH PROTOCOL BY AN INSTITUTIONAL REVIEWER BOARD

The study was reviewed and approved by Clinical Research Ethical Committee of Kensho‐kai Medical Corporation (No. 1201‐05‐005708).

## INFORMED CONCENT

The patients/participants provided their written informed consent to participate in this study.

## REGISTRY AND THE REGISTRATION NO. OF THE STUDY/TRIAL

N/A.

## ANIMAL STUDIES

N/A.

## Supporting information

Supinfo

Supinfo

Supinfo
